# Phenylboronic acid-modified hollow silica nanoparticles for dual-responsive delivery of doxorubicin for targeted tumor therapy

**DOI:** 10.1093/rb/rbw045

**Published:** 2017-01-11

**Authors:** Ling Huang, Qingfeng Zhang, Liangliang Dai, Xinkun Shen, Weizhen Chen, Kaiyong Cai

**Affiliations:** Key Laboratory of Biorheological Science and Technology, Ministry of Education College of Bioengineering, Chongqing University, Chongqing 400044, P. R. China

**Keywords:** hollow mesoporous silica nanoparticles, dual-response, drug delivery, targeted tumor therapy, *in vivo*

## Abstract

This work reports a multifunctional nanocarrier based on hollow mesoporous silica nanoparticles (HMSNs) for targeting tumor therapy. Doxorubicin (DOX) was loaded into HMSNs and blocked with cytochrome C conjugated lactobionic acid (CytC–LA) via redox-cleavable disulfide bonds and pH-disassociation boronate ester bonds as intermediate linkers. The CytC–LA was used both as sealing agent and targeting motif. A series of characterizations demonstrated the successful construction of the drug delivery system. The system demonstrated pH and redox dual-responsive drug release behavior *in vitro*. The DOX loading HMSNs system displayed a good biocompatibility, which could be specifically endocytosed by HepG2 cells and led to high cytotoxicity against tumor cells by inducing cell apoptosis. *In vivo* data (tumor volume, tumor weight, terminal deoxynucleotidyl transferase dUTP nick end labeling and hematoxylin and eosin staining) proved that the system could deliver DOX to tumor site with high efficiency and inhibit tumor growth with minimal toxic side effect.

## Introduction

Tumor is one of the most severe disease that causing human death worldwide [1]. Although conventional therapies (chemotherapy and radiotherapy, etc.) could treat tumors to some degrees, it was also proved that those therapies had severe damage or toxicity against healthy organs [2], due to being lack of cell specificity. Thus, it is urgent for the development of stimuli-responsive controlled-release system for targeted intracellular drug delivery in respect to tumor therapy [3, 4].

Diverse nanocarriers, such as micelles [5], inorganic nanoparticles [6, 7], polymers [8], nanocapsules [9], dendrimers [10] and yolk–shell nanoparticles [11], were developed for drug delivery and biomedical applications. Nevertheless, hollow mesoporous silica nanoparticles (HMSNs) [12, 13] become to be one of the most promising drug nanocarriers for smart drug release delivery, because of its notable advantages, including high drug loading capacity, large surface area, good biocompatibility and easy to be functionalized.

To reduce toxic side effect, the design of cell-specific stimuli-responsive drug delivery system is essentially important. Previously, external stimuli including light [14, 15], temperature [16] and magnetic field [17] were employed to trigger drug release from diverse systems. What’s more, internal signals in tumor microenvironment [18, 19] could be used as stimuli for the design of drug delivery system, since tumor microenvironment contains many signals (pH, redox state, enzymes, etc.) that quite differ from those of surrounding healthy tissues. Many drug delivery systems based on single signal deriving from tumor microenvironment had been exploited, including redox state [12, 20], pH [10, 21], enzymes [22], and so on. Nevertheless, a single stimulus was limited by its low efficiency for triggering drug delivery in some cases. Thus, to exploit dual-responsive drug delivery systems attracted much attention in recent years [23, 24].

As for HMSNs type dug nanocarriers, how to effectively seal their mesopores is essentially important so as to avoid drug leaking on the way to tumor site, which in turn reducing the toxic side effect. Previously, biomacromolecules of heparin [25], bovine serum albumin [26] and collagen [27] were used as end-capping agents for MSNs systems. Cytochrome C (CytC), an electron transfer in oxidative metabolism, was proved to be biocompatible. The global dimensions of CytC are 2.5 × 2.5 × 3.7 nm [28], which makes it an ideal sealing agent for HMSNs. Moreover, CytC is an important mediator of apoptosis by recruiting and activating caspase via conjugation with Apaf-1 in the presence of dATP, once it released from cell mitochondria to cytoplasm [29, 30].

Furthermore, to improve tumor therapy effect, targeted intracellular drug delivery is also essentially important [18]. Comparing with passive targeting via enhancing permeability and retention (EPR) effect, active targeting through targeting motifs including antibody [31], DNA aptamer [32], phenylboronic acid [33], peptide [8] and folic acid [10] could highly enhance the therapeutic effect against tumor and reduce toxic side effect of the system. Previously, lactobionic acid (LA) was proved to be a good targeting agent [12, 25] in respect to HepG2 cells, since it is a specific ligand binding to asialoglycoprotein receptor (ASGP-R) [34] of HepG2 cells.

Herein, we report an approach for the fabrication of multifunctional dual-responsive HMSNs drug delivery system, which was constructed by employing lactobionic acid conjugated cytochrome C (CytC–LA) as gatekeeper to block HMSNs, and redox-cleavable disulfide bonds and pH-disassociation boronate ester bonds as intermediate linkers. When the DOX loading system reaches tumor site via targeting, the overexpressed glutathione (GSH) and acidic pH in tumor microenvironment would break down the intermediate linkers [35–37], leading to rapid drug release against tumor growth. Thus, we hypothesized that the HMSNs system could cell specifically deliver drug to tumor cells in response to GSH and acidic pH stimuli for tumor growth inhibition.

## Experimental

### Materials

Tetraethoxysilane (TEOS), N-hydroxysuccinimide (NHS), DOX hydrochloride, ethyl-3-(3-dimethylaminopropul)carbodiimide hydrochloride (EDC), fluorescein isothiocyanate (FITC), DAPI and rhodamine–phalloidin were purchased from Sigma-Aldrich (Beijing, China). GSH, 3-(triethoxysilyl) propylsuccinic anhydride (95%) and 4-carboxyphenylboronic acid (CPA, 98%) were supplied by J&K Scientific Ltd. LA (97%) was bought from Aladdin Industrial Co. (Shanghai, China). CytC from equine heart ( >95%) was obtained from Solarbio (Beijing, China). Cystamine dihydrochloride (Cys·HCl) and N-cetyltrimethylammonium bromide (CTAB) were provided by Alfa Aesar (Tianjin, China). Cy3-labeled terminal deoxynucleotidyl transferase dUTP nick end labeling (TUNEL) and Annexin V-FITC/PI apoptosis assay kit were purchased from Neobioscience (Shenzhen, China).

### Preparation of HMSNs

HMSNs were synthesized according to previous reports with slight change [12, 13]. SiO_2_ nanoparticles (∼80 nm) were synthesized via Stoeber method. Next, SiO_2_@CTAB–SiO_2_ core/shell nanoparticles were synthesized. Ammonium hydroxide (2.75 ml) and CTAB (0.75 g) were dissolved into 300 ml mixture solution containing water/ethanol (1:1, v/v). The synthesized SiO_2_ nanoparticles (0.5 g) were dispersed into distilled water (100 ml) and added to above solution. After that, TEOS (0.75 ml) was added and stirred for another 6 h. The resulting product (SiO_2_@CTAB–SiO_2_) was collected via centrifugation (11 000 rpm). To selectively etch the SiO_2_ core, sodium carbonate (Na_2_CO_3_, 2.35 g) dissolving in water (25 ml) was added to suspension of SiO_2_@CTAB–SiO_2_ in water (75 ml) and reacted under stirring at 50°C for 8 h. Furthermore, to remove CATB surfactant, the obtained nanoparticles were dispersed into methanol/hydrochloric acid (37.4%) mixture solution (250 ml/15 ml, v/v) and then refluxed at 80°C for 36 h. Finally, the product was rinsed with double-distilled water and methanol each for 3 times and dried under high vacuum (< 1000 Pa) at ambient temperature. The resulting product was named as HMSNs.

### Synthesis of LA-modified CytC 

LA grafting CytC was synthesized using EDC/NHS as coupling agents [37]. First, LA (50 mg) was dissolved into PBS (10 ml, pH 5.0) containing NHS (15 mg) and EDC (30 mg) under stirring. And then, CytC (100 mg) was added to above solution and stirred for another 36 h. Finally, the resulting product was purified with a dialysis tube (8000–14 000 MWCO) against distilled water for 2 days. The resulting product (CytC–LA) was dried by lyophilization.

### Fabrication of HMSNs-based drug delivery system

#### Synthesis of carboxyl-functionalized HMSNs

First, HMSNs (1 g) were dispersed into toluene (100 ml) under stirring for 30 min, and then 3-(triethoxysilyl)propylsuccinic anhydride solution (0.814 ml, 2 mM) was slowly dropped into the above solution. The mixture solution was refluxed at 75°C for 36 h. The nanoparticles were obtained by centrifugation at 11 000 rpm for 8 min, followed by washing with methanol for 3 times. The product was dried under high vacuum (< 1000 Pa) for 24 h and donated as HMSNs–COOH.

#### Synthesis of disulfide linking HMSNs

The disulfide bonds linking HMSNs (HMSNs–S–S–NH_2_) were synthesized as follows [25]. Briefly, the HMSNs–COOH (0.1 g) were dispersed into a mixture solution containing EDC (0.015 M) and NHS (0.015 M) in PBS (20 ml, pH 5) and then Cys·HCl (1 g) was added to the above solution. The resulting solution was stirred at room temperature for 24 h. HMSNs–S–S–NH_2_ were obtained by centrifugation (11000 rpm, 8 min) and then washed with ethanol and water each for 6 times. The product was then dried under high vacuum for further use.

#### Synthesis of CPA-modified HMSNs

Typically, HMSNs–S–S–NH_2_ (0.4 g) was dispersed into DMSO (20 ml). Then, CPA (0.15 g, 0.9 mmol) was dissolved into DMSO (5 ml) containing NHS (0.10 g, 0.87 mmol) and EDC (0.20 g, 1.04 mmol). After stirring at room temperature for 15 min, the mixture solution was added to the HMSNs–S–S–NH_2_ suspension. The mixture solution was stirred at room temperature for another 24 h, followed by centrifugation (11 000 rpm, 8 min) and washing with DMSO, water and methanol, each for 3 times. The sample was donated as HMSNs–S–S–CPA.

#### Construction of HMSNs drug delivery system (HMSNs–S–S–CPA–CytC–LA)

DOX and/or FITC was employed as model drugs in this study. HMSNs–S–S–CPA (36.35 mg) were suspended in PBS (60 ml, pH 7.4) containing DOX (10 mg) or FITC (10 mg) and stirred at room temperature for 24 h. The pH value of above solution was adjusted to 8. After that, CytC–LA (0.2 g) was added and stirred for another 24 h. The resulting functionalized nanoparticles were collected by centrifugation and washed with distilled water for 3 times. The sample was donated as HMSNs–S–S–CPA–CytC–LA@DOX or HMSNs–S–S–CPA–CytC–LA@FITC. To measure the drug loading of the system, calibration curve of DOX was constructed with UV-vis spectrophotometer (NanoDrop 2000c, Thermo) at a wavelength of 480 nm. The drug loading degree (DLD) and drug loading efficiency (DLE) were calculated using the following equations:

$$\begin{array}{c} \text{DLD}\;\left(\%\right)=\left[\text{DOX}\;\text{weight}/\left(\text{HMSNs}-S-S-\text{CPA}-\text{CytC}-\text{LA}\;\text{weight}+\text{DOX}\;\text{weight}\right)\right]\times 100\%;\\ \text{DLE}\;\left(\%\right)=\left[\left(\text{Total}\;\text{DOX}\;\text{weight}\;–\;\text{DOX}\;\text{weight}\;\text{in}\;\text{supernatant}\right)/\text{total}\;\text{DOX}\;\text{weight}\right]\times 100\%. \end{array}$$

### Materials characterization

The morphologies of HMSNs and HMSNs–S–S–CPA–CytC–LA were characterized by transmission electron microscopy (TEM, LIBRA 200 CS; Carl Zeiss Co., Germany) and scanning electron microscopy (FEI-SEM, Nova 400; USA). Surface areas and pore sizes distribution of various HMSNs were characterized by Brunauer–Emmett–Teller (BET) and Barrett–Joyner–Halenda (BJH) (ASAP2020M; USA) analyses, respectively. The potential and dimension distribution of different samples were characterized by Zeta potential measurements (Nano ZS90 Zetasizer; Malvern Instruments Co. Ltd, UK) with dynamic light scattering (DLS) at 25°C. Fourier transform infrared spectroscopy (FTIR, model 6300; Bio-Rad Co. Ltd, USA) and thermogravimetric analysis (TGA, DTG 60H; Japan) were employed to monitor the functionalization procedures.

### Redox and pH dual-responsive drug release

Static and dynamic DOX release profiles of HMSNs–S–S–CPA–CytC–LA @DOX were monitored by UV-vis spectrophotometer as aforementioned. Briefly, HMSNs–S–S–CPA–CytC–LA@DOX (1 mg) was separately dispersed into PBS (2 ml) in different groups (pH 7.4, pH 5.0, pH 7.4 + GSH 2 mM, pH 7.4 + GSH 10 mM and pH 5.0 + GSH 10 mM) in centrifuge tubes and incubated at 37°C under stirring. Then, the solutions were centrifuged at 11 000 rpm for 8 min at given time intervals and 0.1 ml of supernatant liquid was taken out for measuring the drug concentration. Equal volume of fresh PBS was added. The concentrations of drug release were averaged with three measurements in each group.

The corrected concentration of released DOX was calculated as follows [38]:

$$\text{Co}=\text{Ct}+\frac{v}{V}\sum_{0}^{t-1}\text{Ct}$$

where Co means the corrected concentration, Ct is the measurement value of the concentration at time t, v is the volume of sample taken (0.1 ml) and V is the total volume of the fluid (2 ml).

### *In vitro* study

#### Cell culture

Human liver cancer cells (HepG2) and human umbilical vein endothelial cells (HUVECs) were incubated with RPMI1640 medium containing 10% (v/v) fetal bovine serum (Gibco), 100 mg mL ^−^ ^1^ streptomycin and 100 U mL ^−^ ^1^ of penicillin, under a humid atmosphere of 5% CO_2_ at 37°C.

#### Cytotoxicity assay

The cytotoxicity of different HMSNs was evaluated with HepG2 cells using CCK-8 assay [39]. HepG2 cells were seeded in a 24-well plate at 20 000 cells per well and cultured until the cell confluence reaching about 70%. The cells were washed with PBS and then co-cultured with various samples (HMSNs, HMSNs–S–S–CPA–CytC and HMSNs–S–S–CPA–CytC–LA) at a concentration of 0.136 mg/mL for 6, 12 and 24 h, respectively. After that, the medium was replaced by medium composing of 20 μL of CCK8 solution and 200 μL of RPMI1640 and incubated for another 2 h. Finally, the optical absorbance of the culture medium was measured with a microplate reader (Bio-Rad 680; USA). The concentration dependent cytotoxicity of DOX (0.3125, 0.625, 1.25, 2.5 and 10 µg/mL) and HMSNs–S–S–CPA–CytC–LA@DOX (4.3, 8.5, 17.0, 34.0 and 68.0 µg/mL) was also characterized by the same way with a culture time of 36 h.

#### Intracellular nanoparticles distribution

TEM and confocal laser scanning microscopy (CLSM) were employed to reveal the distributions of nanoparticles in cells. Cell seeding procedure was the same as above. Cells were co-cultured with HMSNs and HMSNs–S–S–CPA–CytC–LA (0.136 mg/mL) for 12 h. Cells culturing in tissue culture polystyrene (TCPS) without adding any nanoparticles was used as control. TEM samples were prepared as previous reports [24, 25]. Cells were collected and fixed with glutaraldehyde (2% w/v) and paraformaldehyde (2% w/v) at 4°C for 2 h. Then, the samples were immersed into osmic acid (2%) for 15 min and stained with a uranyl acetate solution for another 15 min. The samples were dehydrated in a graded series of ethanol, followed by incubated with a mixture solution of dehydrated ethanol and Spurr's medium (1:1, v/v) for another 1 h. The samples were dried, cut into ultrathin sections and stained with uranyl acetate on the grid for 5 min. The images were recorded by a TEM microscope. For CLSM imaging, HMSNs@FITC and HMSNs–S–S–CPA–CytC–LA@FITC (0.136 mg/mL) were co-cultured with HepG2 cells for 6, 12 and 24 h, respectively. Next, cells were fixed with para-formaldehyde (4% w/v) at 4°C for 40 min. After that, the treated cells were stained by DAPI (20 µg/mL) and then washed 3 times with PBS. Fluorescence images of cells were acquired by CLSM (LSM 510 META; Olympus, Japan).

#### Cells apoptosis imaging

Cell seeding procedure was the same as above. After that, DOX (20 µg/mL), HMSNs@DOX and HMSNs–S–S–CPA–CytC–LA@DOX (0.136 mg/mL, equal DOX content) were added and cultured for 6, 12 and 24 h, respectively. The cell staining method was similar to the above, except for rhodamine–phalloidin was added and incubated at 4°C overnight before staining with DAPI. Finally, the cells were observed by CLSM. The dual fluorescence of Annexin V-FITC/PI by CLSM was further employed to analyze cell apoptosis according to the kit instructions.

#### Flow cytometry

Flow cytometry (FC) was utilized to monitor the endocytosis efficiency of FITC-labeled nanoparticles. HepG2 cells and HUVECs were cultured into a six-well plate at an initial seeding density of 30 000 cells cm ^−^ ^2^, respectively. Cells were then incubated with HMSNs–S–S–CPA–CytC–LA@FITC (0.136 mg/mL) for 2 and 4 h, respectively. Finally, cells were collected and fixed with binding buffer for analysis by a FACS Calibur (BD Biosciences).

To investigate cell targeting property of the system, HepG2 cells were pre-treated with LA or not. Equal amount of HMSNs–S–S–CPA–CytC–LA@FITC (0.136 mg/mL) were added and co-cultured with cells for another 24 h. FC analysis was performed as the same above. FC analysis was also used to measure cell apoptosis induced by DOX, HMSNs@DOX or HMSNs–S–S–CPA–CytC–LA@DOX with an annexin V-FITC kit. HepG2 cells were seeded at a density of 20 000 cells per cm^2^. DOX (20 µg/mL), HMSNs, HMSNs@DOX and HMSNs–S–S–CPA–CytC–LA@DOX (0.136 mg/mL) were added and incubated at 37°C for 24 h. Cells were collected and re-suspended in binding buffer at a concentration of 10^6^ cells per mL. Then, 5 ml of FITC conjugated annexin V and 10 ml of propidium iodide were added to cell suspension (195 ml) and incubated at room temperature for 15 min in dark. Finally, 300 ml of supernatant was taken out and analyzed using a FACS Calibur.

### *In vivo* evaluations

#### Tumor model

Male nude mice (average weight of 19.87 ± 0.95 g) were bought from Xinqiao Hospital (Chongqing, China). All *in vivo* animal experiments were operated in compliance with the animal management rules of the ethics committee of Third Military Medicine University (Chongqing, China). All mice were tenderly treated by subcutaneously injected HepG2 cells at a concentration of 10^6^ cells per 100 µL at the groin on one side of each mouse. When tumors reached an average volume of 50 mm^3^, the tumor-bearing mice were subjected to tumor therapy experiments. The tumor volumes of nude mice were monitored by a digital caliper and calculated by a formula: (Tumor volume = (tumor length) × (tumor width)^2^/2).

#### In vivo tumor therapy

The mice were randomly divided into five groups (four mice per group) with the similar tumor volume and body weight. The samples (saline, HMSNs, HMSNs@ DOX, DOX and HMSNs–S–S–CPA–CytC–LA@DOX) were injected into the mice via tail vein. The DOX amount was defined as 3 mg kg ^−^ ^1^ d ^−^ ^1^. Tumor size and mice weight were recorded every 2 days.

#### Immunohistochemistry staining

All mice were sacrificed after treatments for 20 days. Kidney, liver, tumor, lung heart and spleen of mice were taken out and fixed with 10% formalin at 4°C for 48 h. After that, all organs and tumor tissue were embedded in paraffin, sectioned and stained with H&E. Optical microscope was applied to observe the histological sections.

#### TUNEL staining

Tumor biopsies were stained with a TUNEL apoptosis kit (Beyotime Co.). Briefly, tumor biopsies were de-paraffinized; hydrated with dimethyl benzene and ethanol; and treated with protease K at 37°C for 30 min. After that, the samples were washed with PBS for 3 times. Biopsies were incubated with 50 µL of TUNEL solution in dark at 37°C for 1 h. Finally, DAPI (20 µg/mL) solution was added and incubated at room temperature for 10 min. The biopsies were observed by CLSM.

### Statistical analysis

All data were presented as means  ±  standard deviation (SD). OriginPro (version 8.0) was utilized and statistically analyzed via student’s *t*-test and the one-way analysis of variance in this study. The confidence levels of 95% and 99% were regarded as significant difference.

## Results and Discussion

### Synthesis and characterization of HMSNs-based drug delivery system

**Figure 1:**
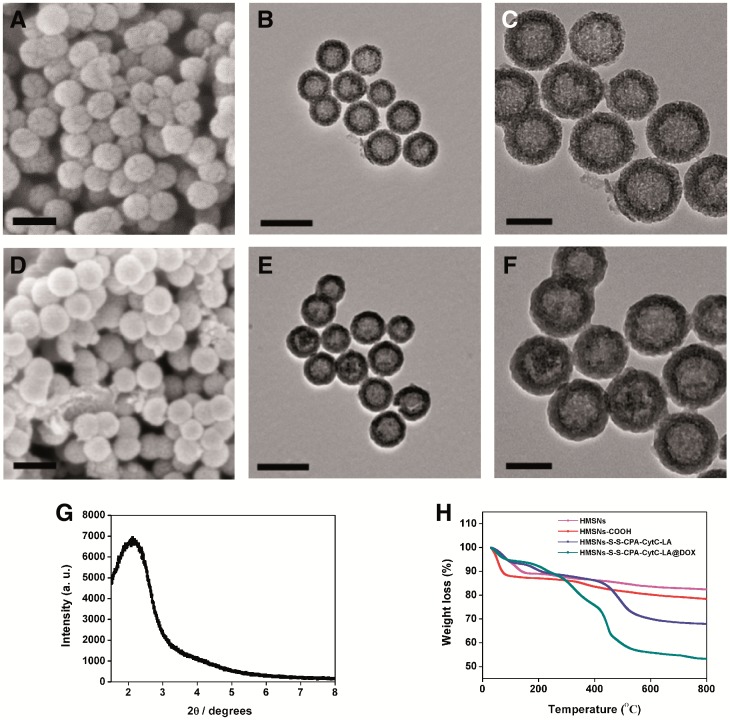
Physical property characterizations: SEM and TEM images of HMSNs (A and B) and HMSNs–S–S–CPA–CytC–LA (D and E) nanoparticles with corresponding high resolution TEM images (C and F). The scale bars were 200 nm for (A), (B), (D) and (E) and 100 nm for (C) and (F); (G) low-angle XRD pattern of HMSNs; (H) TGA curves of various functionalized HMSNs

Briefly, HMSNs was synthesized [12, 13], carboxyl-functionalized and coupled with disulfide bonds via amidation reaction with Cys·HCl. The 4-carboxyphenylboronic acid was then conjugated to the system again with amidation reaction. Finally, CytC–LA was grafted onto the HMSNs system through boronate ester bonds (Fig. S1, see online supplementary material for a colour version of this figure), which was sensitive to the acidic stimulus. SEM and TEM images show that HMSNs demonstrated round spherical features with distinct hollow cavity and shell structures (Fig. 1A–C). The synthesized HMSNs displayed relatively good dispersion. After multi-functionalization with different molecules step by step, the resulting HMSNs–S–S–CPA–CytC–LA nanoparticles displayed similar morphology and structure to those of HMSNs. However, a reduction contrast regarding mesopores and unclear shell layer circling the HMSNs were observed (Fig. 1D–F;Figures S2 and 3, See online supplementary material for a colour version of this figure), which was related to intermediate linkers bonding and immobilization of CytC–LA onto the surfaces of HMSNs. Small angle XRD analysis displayed an obvious peak at 2.09° (Fig. 1G), implying the as-synthesized HMSNs had highly ordered lattice array and distinct mesoporous structure. The result was consistent with a previous study [40]. Moreover, DLS measurements revealed that both HMSNs and HMSNs–S–S–CPA–CytC–LA had relatively narrow size distributions, with average sizes of 105.4 and 115.8 nm (Fig. S4, see online supplementary material for a colour version of this figure), respectively. Previous studies confirmed that nanoparticles with dimensions below 200 nm could be well circulated *in vivo* and endocytosed with high efficiency for drug delivery [4]. Thus, the obtained HMSNs system could be used as a promising drug vehicle.

To validate the successful fabrication of the HMSNs-based drug delivery system, Fourier transform infrared spectroscopy (FTIR), thermogravimetric analysis (TGA), BET, BJH analysis and zeta potential measurements were employed to characterize the modification processes. The FTIR spectra indicated the successful functionalization of HMSNs with various molecules step by step (Fig. S5, see online supplementary material for a colour version of this figure). Furthermore, TGA curves showed the weight loss of HMSNs system corresponding to the sequential modification processes. About 14.6 wt% of DOX was loaded into the HMSNs–S–S–CPA–CytC–LA (Fig. 1H), which was much higher than that of traditional MSNs (less than 10 wt%) [20, 24]. Moreover, the DLD and DLE of HMSNs–S–S–CPA–CytC–LA@DOX system were calculated to be around 14.73 wt% and 62.8%. The DLD result was slightly higher than that of TGA measurement, which was related to the physical absorption of DOX on the surfaces of multi-functionalized HMSNs.

Moreover, nitrogen adsorption–desorption isotherms of different HMSNs were measured. The samples displayed a type IV isotherm with type H1 hysteresis loops and characteristics of mesoporous material with 1D cylindrical channel [38]. Furthermore, the isotherm exhibited considerable changes after coupling with different functional units. The BET surface areas of HMSNs system decreased from 881.54   to 90.35 m^2^ g ^−^ ^1^ along with the sequential modifications (Fig. S6A (see online supplementary material for a colour version of this figure) and Table S1). The same trend was also observed for BJH pore diameters (Fig. S6B (see online supplementary material for a colour version of this figure) and Table S1). The results suggest that the mesopores (∼4.0 nm) of HMSNs were successfully sealed with CytC–LA molecules.

Besides, zeta potentials of various samples were measured to reveal the functionalization processes (Table S2). After carboxylation, the zeta potential of HMSNs–COOH decreased from −23.6 to −30.9 mV, which was attributed to the introduction of negatively charged carboxyl groups. For HMSNs–S–S–NH_2_, the zeta potential increased from −30.9 to 18.2 mV, which was contributed to the conjugation of positively charged Cys·HCl molecules. After modification with CPA and CytC–LA, the zeta potentials shifted to 2.85 and -24.3 mV. The data again suggest that HMSNs–S–S–CPA–CytC–LA was successfully fabricated.

### Redox and pH dual-responsive drug release

**Figure 2:**
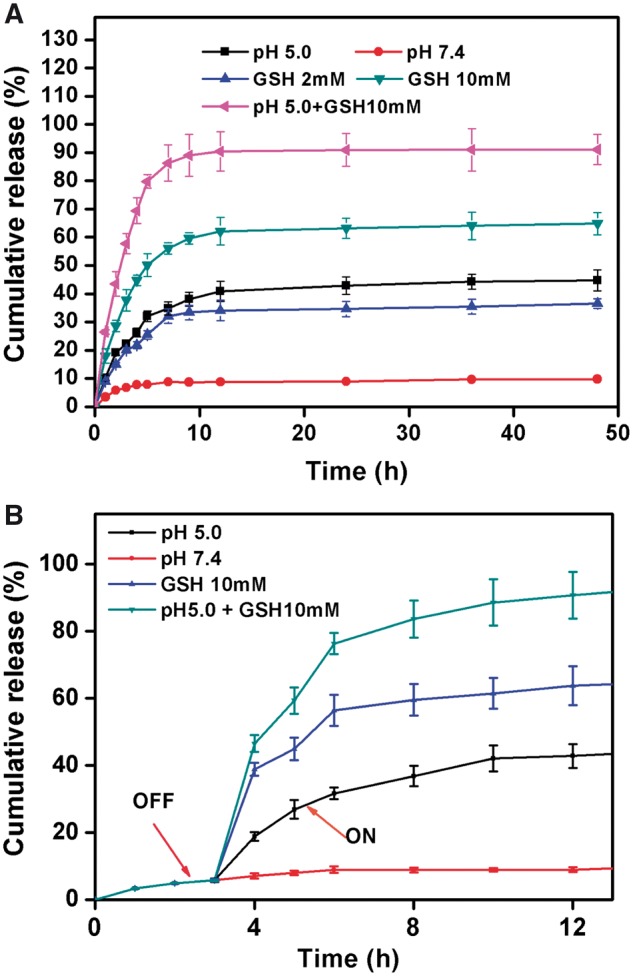
(A) Dual-responsive DOX release from HMSNs-S-S-CPA-CytC-LA@DOX with various stimuli and (B) dynamic drug release behavior of the system over 12 h

To investigate real-time release behavior of HMSNs–S–S–CPA–CytC–LA@DOX, redox and pH stimuli were employed in this study (Fig. 2A). Under physiological condition (pH 7.4), only a negligible amount of DOX (< 10% over 24 h) released from HMSNs–S–S–CPA–CytC–LA@DOX, indicating good sealing efficiency of the system. However, relatively high amount of DOX (around 40% over 24 h) released from the system when exposing to acidic environment (pH 5.0), indicating its pH sensitivity. It was attributed to the disassociation of boronate ester bonds under acidic environment [35–37].

To investigate redox-responsive drug release behavior, GSH was used as a stimulus to trigger drug release from the system. Around 33 and 62% of DOX released when the system was exposed to GSH with concentrations of 2 and 10 mM, respectively. It could be explained that GSH broken down the disulfide linkage of the system. Considering acidic condition of tumor microenvironment and abundant distribution of GSH in the cytoplasm of tumor cells [4, 18, 20], the HMSNs–S–S–CPA–CytC–LA@DOX system demonstrate itself great feasibility for tumor therapy. To further simulate the intracellular environment of tumor cells, the HMSNs–S–S–CPA–CytC–LA@DOX system was dispersed into the PBS (pH 5.0) mixing with GSH (10 mM), resulting in rapid drug release of about 89% in 12 h. It was related to the simultaneous breakage of disulfide bonds and disassociation of boronate ester bonds of the system. The DOX release behavior from the system with various treatments was also clearly observed by optical images (Fig. S7, see online supplementary material for a colour version of this figure).

A drug delivery system triggering by single stimulus, could only achieve certain efficiency for delivering drug to tumor cells. When tumor cells endocytosing a large amount of drug carrier at the same time, single stimulus (e.g. pH or redox) is not sufficient for triggering all rapid drug release into cytoplasm. Under such circumstance, partial drug carriers would be excreted out of cells eventually [40]. Thus, a dual-responsive drug delivery system has advantages for improving the drug delivery efficiency and increase the drug utilization efficiency for tumor therapy. In the present study, the results suggested that the HMSNs–S–S–CPA–CytC–LA@DOX system could be triggered by dual stimuli of pH and GSH for rapid drug release and much higher drug release efficiency (> 89%) as comparing with that of single stimulus.

Furthermore, the dynamic drug release behavior of the system was investigated to simulate the clinical application *in vivo*. To ideally simulate the clinical application for tumor therapy, the microenvironment was adjusted to pH 5.0, 10 mM GSH and pH 5.0 PBS buffer mixing with 10 mM GSH, after incubation for 3 h. Around 42, 63 and 89% of DOX released from HMSNs–S–S–CPA–CytC- LA@DOX system after further incubation for 9 h (Fig. 2B), which suggesting the feasibility of the system for clinical tumor therapy. In details, the end-capping agent of CytC–LA effectively blocked the mesopores of the HMSNs, preventing DOX leakage from the system on the way for tumor site, which would take several 10 min or hours. The system reaches tumor site via the EPR effect and cell targeting. Once the system reached the tumor site, the over expressed reducing agents (e.g. GSH) and acidic environment (e.g. lysosome) within the tumor microenvironment broke down the –S–S– linkers and boronate ester bonds of the system, leading to the rapid release of DOX from the system for killing tumor cells.

### Cytocompatibility and apoptosis assays

**Figure 3:**
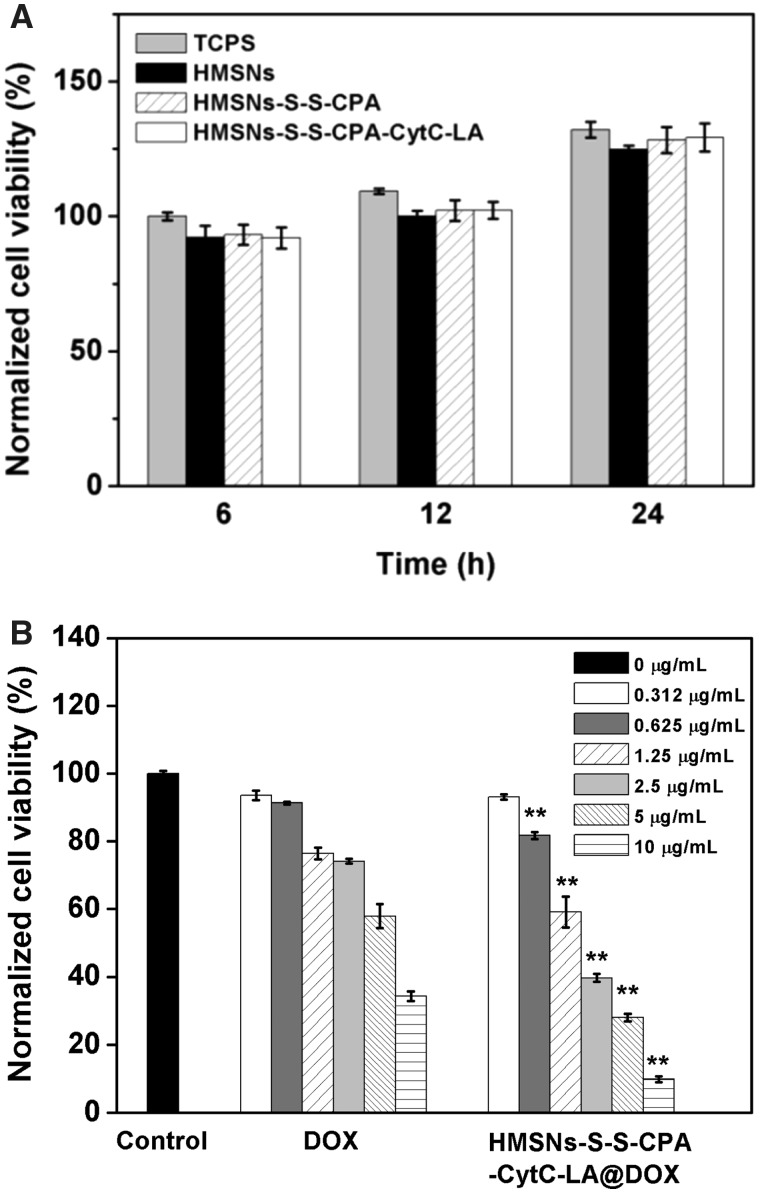
*In vitro* cytotoxicity assays: (A) normalized cell viability of HepG2 cells co-cultured with various functionalized HMSNs in comparison to the control (TCPS) after incubation for 6, 12 and 24 h; (B) concentration dependent cytotoxicity assay of free DOX or HMSNs–S–S–CPA–CytC–LA@DOX after incubation for 36 h. The statistical analysis was performed by comparing the corresponding concentration of free DOX. Error bars reflect mean  ±  SD (*n*  =  4), **P  <  0.01

CCK-8 was applied to evaluate the potential cytotoxicity of various functionalized HMSNs and the inhibitory effect of DOX-loaded HMSNs on HepG2 cells growth. After incubation with medium containing HMSNs, HMSNs–S–S–CPA–CytC and HMSNs–S–S–CPA–CytC–LA for 6, 12 and 24 h, no significance difference of cells viability was observed when comparing with that of cells cultured on TCPS plates (control) at different time intervals (Fig. 3A), indicating the good cytocompatibility of different HMSNs.

Moreover, we performed the dose-dependent cytotoxicity assay of HMSNs–S–S–CPA–CytC–LA@DOX system. The results showed that low concentration (< 0.625 µg mL^−^ ^1^ of DOX) of nanoparticles could only led to limited cytotoxicity after culture for 36 h, whereas high concentrations of nanoparticles (0.625–10 µg mL^−^ ^1^ of DOX) resulted in severe cytotoxicity (*P*  <  0.01) in comparison to the corresponding concentration of free DOX (Fig. 3B). The above results could be explained as follows: the HMSNs–S–S–CPA–CytC–LA@DOX system were internalized by HepG2 cells via EPR effects and active targeting endocytosis and then triggered by intracellular acidic environment and overexpressed GSH. As a result, the sustained release of DOX induced relative higher percentage of cell apoptosis than the free DOX.

### *In vitro* cellular uptake and distribution assay

**Figure 4:**
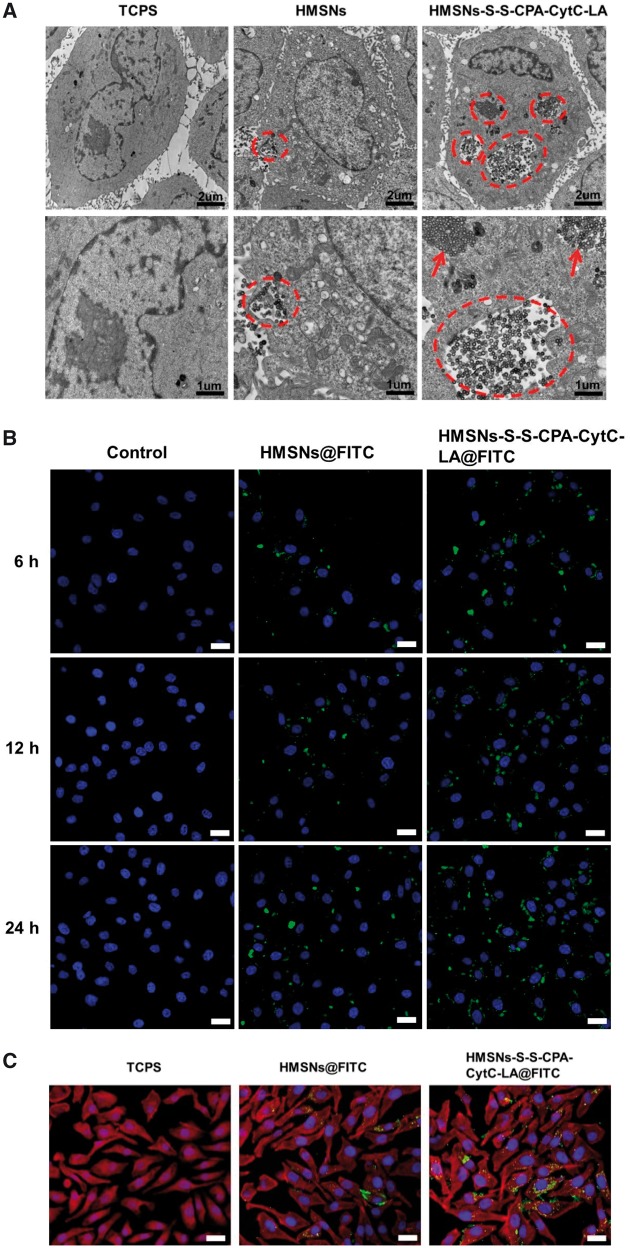
Endocytosis assays: (A) TEM images of various HMSNs distributions in HepG2 cells after incubation for 12 h. The images in bottom row show the magnified view of the endocytosed nanoparticles; (B) CLSM images of HepG2 cells (nuclei) after culture with TCPS, HMSNs@FITC and HMSNs–S–S–CPA–CytC–LA@FITC for different periods. Scale bar: 30 µm. (C) CLSM images showing the nanoparticles distributions in HepG2 cells after culture with TCPS, HMSNs@FITC and HMSNs–S–S–CPA–CytC–LA@FITC for 24 h. Scale bar: 30 µm. Cytomembrane (red), nuclei (blue), nanoparticles (green)

To evaluate the interactions of HepG2 cells with various functionalized HMSNs, TEM and CLSM were employed. TEM was applied to observe the morphology and distributions of HMSNs and HMSNs–S–S–CPA–CytC–LA within HepG2 cells. The images exhibited distinct cell membrane and intact nuclei comparing with those of control (Fig. 4A), which was contributed to the good biocompatibility of HMSNs (Fig. 3A). The endocytosed nanoparticles entered into the cytoplasm rather than the nuclei, which was consistent with previous studies [11, 12, 37]. Interestingly, the uptake amount of HMSNs–S–S–CPA–CytC–LA was much higher than that of HMSNs, which was attributed to the targeting LA molecule specially binding with the ASGP-R on the membrane of HepG2 cells [34]. More importantly, both endocytosed nanoparticles demonstrated good dispersion within cells cytoplasm (Fig. 4A, dashed circles), which was essential for intracellular drug delivery.

CLSM images also confirmed that mostly of the nanoparticles were distributed into the cytoplasm. The amounts of endocytosed nanoparticles gradually increased in both groups as time extending from 6 to 12 and 24 h (Fig. 4B). Additionally, the fluorescence intensity quantification analysis result also revealed that the HepG2 cells endocytosed higher (*P*  <  0.01) amount of HMSNs–S–S–CPA–CytC–LA@FITC than that of HMSNs@FITC at each time of interval (Fig. S7, see online supplementary material for a colour version of this figure). This could be attributed to LA-mediated cell-specific endocytosis. CLSM observation also indicated that HepG2 cells displayed well-spreading morphologies with intact cell nuclei (blue) and regular cell cytoskeletons (red) as compared with the control group (without nanoparticles) after incubation for 24 h (Fig. 4C).

**Figure 5:**
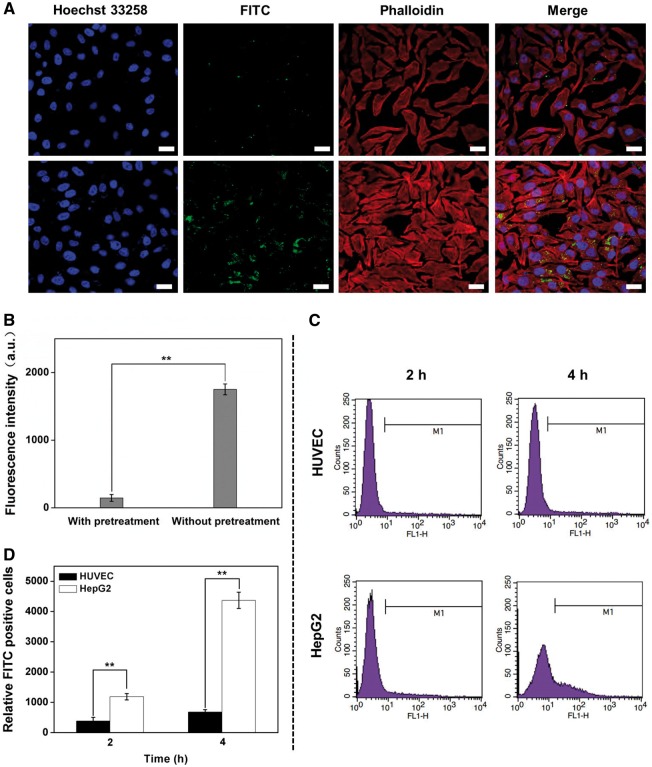
Cell targeting characterization: (A) CLSM images of HepG2 cells pre-cultured with galactose or not (lower row) and then co-cultured with HMSNs–S–S–CPA–CytC–LA@FITC for 24 h. Scale bars: 20 µm. (B) fluorescence quantitative intensity analysis based on above CLSM images; (C) FCM analysis of the endocytosed of HMSNs–S–S–CPA–CytC–LA@FITC by HUVECs (upper row) and HepG2 cells (lower row); (D) quantification analysis corresponding with above FCM analysis. The error bars indicate mean  ±  SD (*n*  =  3), **P  <  0.01

To reveal the LA mediated cell targeting, LA receptors on the HepG2 cells were blocked by pre-incubation with excessive LA before treatment with HMSNs–S–S–CPA–CytC–LA@FITC. HepG2 cells without pre-incubation with free LA showed higher endocytosis efficiency than that of control (Fig. 5A). Fluorescence intensity quantification analysis results also proved the uptake of HMSNs–S–S–CPA–CytC–LA by HepG2 cells was achieved by LA receptor-meditated endocytosis pathway (Fig. 5B). Additionally, HUVECs were employed as control cells to investigate the cell-specific endocytosis with FC (Fig. 5C). The results suggest that HepG2 cells endocytosed significantly higher (*P* < 0.01) amount of FITC labeled nanoparticles than that of HUVECs (Fig. 5D). The significant difference could be explained by the fact that the amount of LA receptors on tumor cells was much higher than that of normal cells, which facilitating specific receptor-mediated endocytosis of HMSNs–S–S–CPA–CytC–LA@FITC by HepG2 cells. Thus, these results indicated that HMSNs–S–S–CPA–CytC–LA@FITC was specifically endocytosed by tumor cells via a receptor–ligand recognition pathway.

### Inhibitory effects assay of HMSNs system *in vitro*

**Figure 6:**
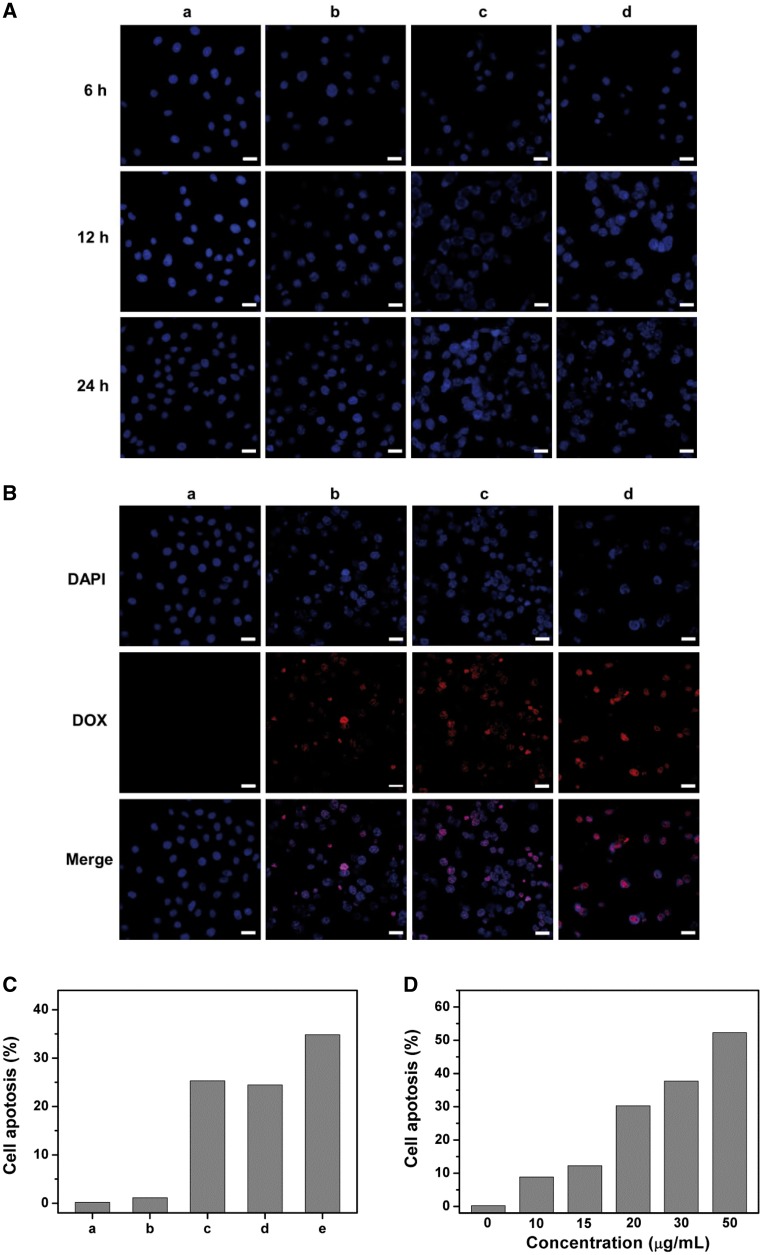
Cell apoptosis analysis *in vitro*: (A) representative CLSM images of HepG2 cells after culture with (a) TCPS, (b) DOX, (c) HMSNs@DOX, (d) HMSNs–S–S–CPA–CytC–LA@DOX with equivalent concentration of DOX (20 µg/mL) for 6, 12 and 24 h, respectively; (B) merged CLSM images from DAPI and DOX channels after incubation with different nanoparticles for 24 h. Blue: nuclei, red: DOX. Scale bar: 30 µm. (C) FCM analysis of HepG2 cell apoptosis induced by different treatments with PBS (a, control), HMSNs (b), DOX (c), HMSNs@DOX (d), HMSNs–S–S–CPA–CytC–LA@DOX (e) for 24 h; (D) FCM analysis of concentration dependent cell apoptosis induced by HMSNs–S–S–CPA–CytC–LA@DOX after culture for 24 h

CLSM and FC were used to evaluate the effect of HMSNs–S–S–CPA–CytC–LA@DOX on cell growth. HepG2 cells cultured onto TCPS displayed oval or round cells nuclei with clear boundaries (Fig. 6A, a), suggesting the cells were in healthy state. However, the cell nuclei became shrank after co-culture with free DOX, HMSNs@DOX and HMSNs–S–S–CPA–CytC–LA@DOX for 24 h (Fig. 6A, b–d), indicating that the cells were in apoptotic state, in particular of HMSNs–S–S–CPA–CytC–LA@DOX group. The level of cell apoptosis in each group increased with increasing culture time. The phenomenon above could be explained that hydrophilic DOX was easily dissolved into culture medium and few amount of DOX was endocytosed by cells, leading to relatively low cell apoptosis level. However, most of the HMSNs–S–S–CPA–CytC–LA@DOX was endocytosed via EPR effects and active targeting. The DOX locally released into cytoplasm triggering by intracellular acidic environment and reducing agents. Thus, the relatively high concentration of DOX accumulated in cytoplasm to result in cell apoptosis. Moreover, the dissociating CytC from the system that distributed into cytoplasm could potentially induce cell apoptosis [29, 30].

The growth of HepG2 cells was severely inhibited after treatments with free DOX, HMSNs@DOX and HMSNs–S–S–CPA–CytC–LA@DOX as comparing with that of control (Fig. 6B). Moreover, it was observed that the cell nuclei presenting a mixed color of red and blue after culture with DOX or DOX-loaded nanoparticles, which could be explained by the fact that free DOX or released DOX from the system could interact with cell nuclei and destroy their double-stranded DNA structures. Additionally, the red intensity within HepG2 cells was different among these treatment groups. The HMSNs–S–S–CPA–CytC–LA@DOX group showed higher red intensity than cells treated with free DOX or HMSNs@DOX (Fig. 6B, d vs b and c). The results above could be interpreted by the reason that CytC–LA could effectively seal DOX in HMSNs–S–S–CPA–CytC–LA@DOX until the system was endocytosed by HepG2 cells, while DOX would be leaked out from HMSNs@DOX during the delivery process.

Next, cell apoptosis analysis was examined with the dual fluorescence of Annexin V-FITC/PI by CLSM (Fig. S9, see online supplementary material for a colour version of this figure). Compared with control, the HMSNs–S–S–CPA–CytC–LA@DOX group showed significant cell apoptosis. Subsequently, FC and correspondingly quantification analysis were employed to further evaluate apoptosis level of tumor cells induced by nanoparticles (Fig. S10, see online supplementary material for a colour version of this figure). The results suggest that HMSNs only induced negligible cell apoptosis (Fig. 6C, b), due to its good biocompatibility (Fig. 3A). However, the free DOX, HMSNs@DOX and HMSNs–S–S–CPA–CytC–LA@DOX groups demonstrated prominent cell apoptosis with 25.31, 24.45, and 34.83% (Fig. 6C, c–e), respectively. The HMSNs–S–S–CPA–CytC–LA@DOX system showed the highest cell apoptosis level among all groups. We also observed that HMSNs–S–S–CPA–CytC–LA@DOX induced cell apoptosis in a dose-dependent manner (Fig. 6D).

### *In vivo* evaluations

**Figure 7:**
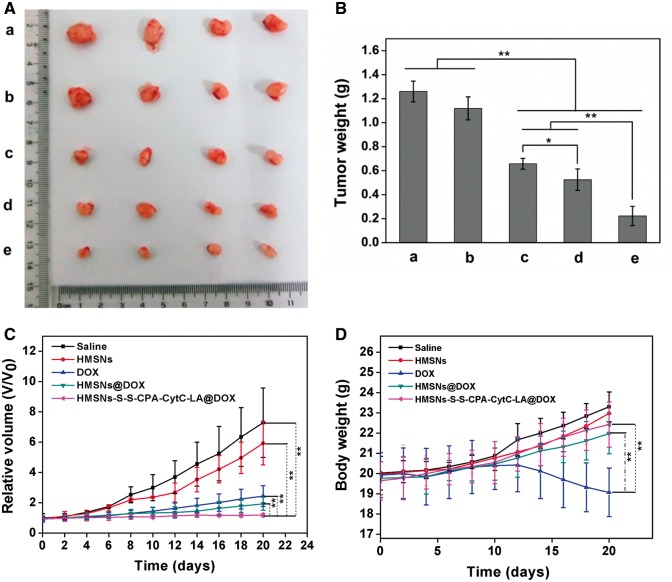
*In vivo* study: (A) representative images of tumors after treatments with saline (a, control), HMSNs (b), DOX (c), HMSNs@DOX (d), and HMSNs–S–S–CPA–CytC–LA@DOX (e) for 20 days, respectively; (B) the final tumor weight of different groups; (C) the changes of tumor volumes with the various treatments; (D) relative body weight of mice subjected to different treatments. The error bars indicate mean ± SD (*n*  =  4), **P  <  0.01, *P  <  0.05

All results above illustrated that HMSNs–S–S–CPA–CytC–LA @DOX was a promising drug delivery system. To evaluate its feasibility for potential tumor therapy, we conducted *in vivo* experiments. After treatments for 20 days, mice were killed, and tumor tissues were collected (Fig. 7A). Obvious tumor inhibition could be observed from the tumor volumes and weights. The tumor weight of mice treated with HMSNs displayed similar to that of saline group (1.18  ±  0.12 g vs 1.26 ± 0.09 g). While the tumor weights of mice treated with DOX (0.66  ±  0.05 g) and DOX-loaded nanoparticles were obviously lower than that of control groups. The HMSNs–S–S–CPA–CytC–LA@DOX-treated group (0.22  ±  0.08 g) demonstrated the lowest tumor weight and smallest tumor size among all the groups (Fig. 7A and B). The results directly imply its great potential for the suppression of tumor growth.

Next, the tumor volumes and mice weight were measured at different time of intervals after injection with saline, HMSNs, DOX and DOX-loaded nanoparticles. Compared with either HMSNs or saline groups, relatively severe tumor inhibitions were observed with treatments by DOX, HMSNs@DOX and HMSNs–S–S–CPA–CytC–LA@DOX. Interestingly, the HMSNs–S–S–CPA–CytC–LA@DOX group showed the greatest potential (*P* < 0.01) of tumor suppression among all groups (Fig. 7C).

The results above suggest fact that DOX could only retain a transient high drug concentration, then quickly distributed into other tissues, which resulted in severe toxic side effects on normal cells and tissues *in vivo*. Moreover, free DOX could be excreted out of body via blood circulation and metabolism and thus resulting in few amount of DOX accumulating into the tumor tissues [41, 42]. To some extent, a blood drug concentration of HMSNs@DOX could be retained at a certain level through sustained release when they were injected. Meanwhile, HMSNs@DOX might relieve some toxic side effects of DOX on the body and still could not specially be delivered to the target tumor tissues. Based on the issues above, HMSNs–S–S–CPA–CytC–LA@DOX system was fabricated, which demonstrated efficient inhibition to the tumor growth. The principle could be illustrated that the CytC sealing units on HMSNs could effectively prevent DOX release when the nanoparticles were circulated in the blood. Furthermore, the LA targeting units could actively promoted HMSNs-S-S-CPA-CytC–LA@DOX to accumulate in tumor tissues. Then the DOX was released into the cytoplasm triggering by intracellular acidic environment and reductive GSH, which could lead to strong tumor suppression. Besides, the dissociated CytC from the system distributing into the cytoplasm could also contribute to cell apoptosis and/or death.

Moreover, the mice weight was also monitored during the therapy process (Fig. 7D). The initial average weight of each group was around 19.87  ±  0.15 g. The mice weight of the group treated with free DOX slightly decreased to 19.075 g after treatments for 20 days. This could be interpreted that DOX randomly diffused into normal tissues/organs via blood circulation, leading to severe toxic side effect. However, the groups treated with saline, HMSNs, HMSNs@DOX and HMSNs–S–S–CPA–CytC–LA@DOX gradually increased to 23.3, 22.97, 21.97 and 22.43 g, indicating that HMSNs–S–S–CPA–CytC–LAhad a good biocompatibility.

**Figure 8:**
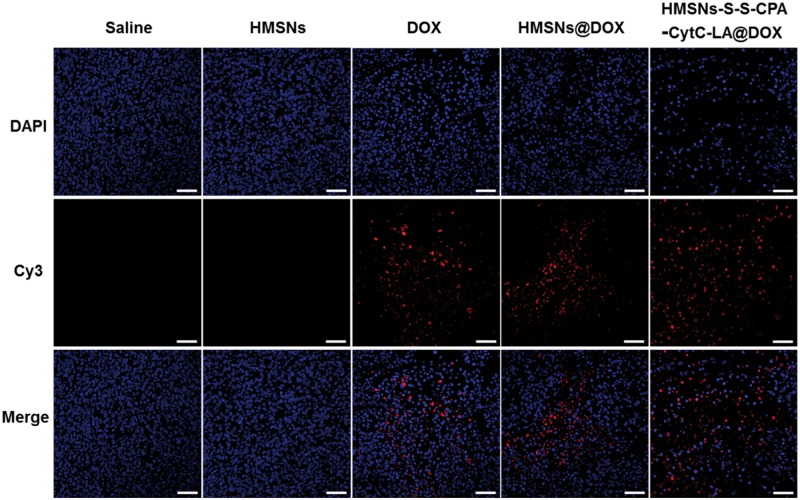
TUNEL Staining of tumors treated with saline, HMSNs, DOX, HMSNs@DOX and HMSNs–S–S–CPA–CytC–LA@DOX for 20 days. Red: apoptotic DNA; blue: cell nuclei. Scale bars: 50 µm

Additionally, histological evaluation of tumor tissues was performed by a TUNEL assay. Saline and HMSNs groups showed negligible cell apoptosis, which was illustrated by the amount of red dots (apoptotic DNA). The phenomenon further demonstrated the good biocompatibility of HMSNs. Although DOX, HMSNs@DOX and HMSNs–S–S–CPA–CytC–LA@DOX groups exhibited obvious cell apoptosis. Interestingly, HMSNs–S–S–CPA–CytC–LA@DOX demonstrated the most severe cell apoptosis (Fig. 8). In addition, the cell nuclei were deformed and cracked. The curative effect of HMSNs@DOX against tumor was better than that of pure DOX group. The results firmly demonstrated that HMSNs–S–S–CPA–CytC–LA@DOX system could efficiently deliver DOX to tumor cells and induce apoptosis *in vivo*, which was consistent with the results above.

**Figure 9:**
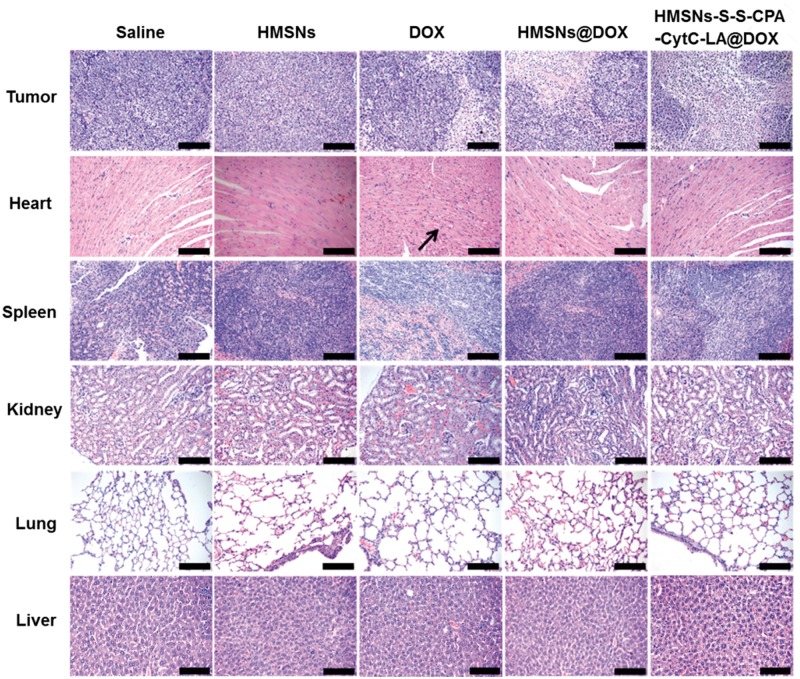
Histological observations of tumors and different organs (heart, liver, kidney, lung and spleen) via H&E staining. Scale bars: 100 µm

Finally, H&E staining of major tissues was employed to illuminate the potential clinical application of the HMSNs–S–S–CPA–CytC–LA@DOX system (Fig. 9). Compared with other groups, HMSNs–S–S–CPA–CytC–LA@DOX group revealed the most severe tumor damage, while no obvious damage against major organs (heart, liver, spleen, lung and kidney). However, DOX group demonstrated a typical myocardial injury when comparing with other groups, which was attributed to the toxic side effects of free DOX [43]. These results suggested that the HMSNs–S–S–CPA–CytC–LA@DOX system could be an ideal vehicle for curing the tumor with high efficiency and limited toxic side effects. Taken together, we confirmed our hypothesis that HMSNs–S–S–CPA–CytC–LA@DOX system could cell specifically deliver drug to tumor site in responding to GSH and pH stimuli for tumor growth inhibition.

## Conclusion

In summary, a redox and pH dual-responsive drug delivery system based on HMSNs was constructed for targeted tumor therapy *in vitro* and *in vivo*. A series of characterizations by combined techniques (e.g. SEM, TEM, BET, BJH, FTIR, TGA, XRD, zeta potential measurement, etc.) confirmed the successful fabrication of the system. The systemic *in vitro* and *in vivo* evaluations demonstrated that the HMSNs–S–S–CPA–CytC–LA@DOX system could be triggered for DOX release by intracellular acidic environment and overexpressed GSH, resulting in cell apoptosis and tumor growth inhibition with high efficiency.

## Supplementary Material

Supplementary Data
