# Supplementary material for: Phenylboronic acid-modified hollow silica nanoparticles for dual-responsive delivery of doxorubicin for targeted tumor therapy
Source: Regen Biomater. 2017 Jan 11;4(2):111–24. doi: 10.1093/rb/rbw045 (PMC6371689; doi:10.1093/rb/rbw045)
Supplement: Supplementary Data [file rbw045_Supp.zip › Supporting Information-Revision.docx]

**Phenylboronic acid-modified hollow silica nanoparticles for dual-responsive delivery of doxorubicin for targeted tumor therapy**

Ling Huang^†^, Qingfeng Zhang^†^, Liangliang Dai, Xinkun Shen, Weizhen Chen, Kaiyong Cai*

† Zhang and Huang contributed equally to this work.

Key Laboratory of Biorheological Science and Technology, Ministry of Education, College of Bioengineering, Chongqing University, Chongqing 400030, China

*Corresponding author: Prof. Kaiyong Cai

College of Bioengineering

Chongqing University

Chongqing 400030

China

Tel: +86-23-65111802

Fax: +86-23-65102877

E-mail: kaiyong_cai@cqu.edu.cn

***List of Contents***

**Figure S1.** Scheme of the fabrication of HMSNs-S-S-CPA-CytC-LA nanocarrier………..S3

**Figure S2.** SEM images of different HMSNs……………………………………………………………..S4

**Figure S3.** TEM images of different HMSNs……………………………………………………………..S5

**Figure S4.** Particles size distributions of different HMSNs………………………………………..S6

**Figure S5.** FTIR spectra of different substances and HMSNs…………………………………….S7

**Figure S6.** BET and BJH analysis…………………………………………………………………………….S10

**Figure S7**. Optical images of DOX release triggered by various stimuli…………….…….S11

**Figure S8.** Quantitative fluorescence intensity analysis……………………………………S12

**Figure S9.** Dual fluorescence of Annexin V-FITC/PI by CLSM………………………………S13

**Figure S10**. Flow cytometry analysis………………………………………………………………………S14

**Table S1.** BET and BJH parameters of different HMSNs..………………………………………..S15

**Table S2.** Zeta-potentials of different HMSNs………………………………………………….…….S16

**References for Supporting Information** ………..…………………………………….…………...…S17


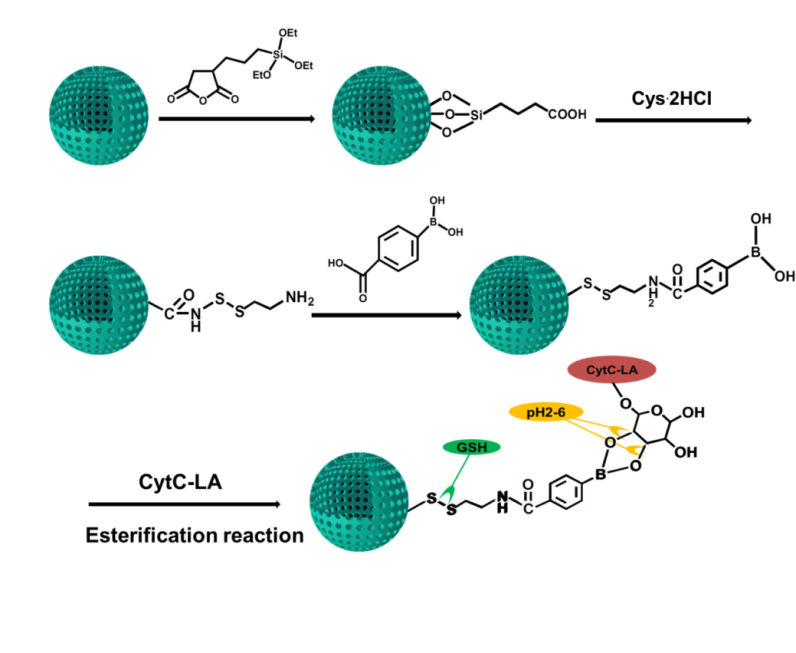


**Figure S1**. Scheme of the fabrication of HMSNs-S-S-CPA-CytC-LA nanocarrier.


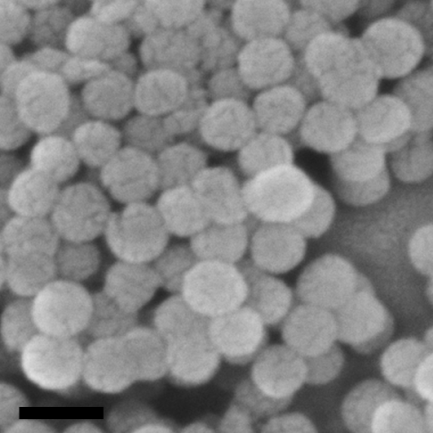


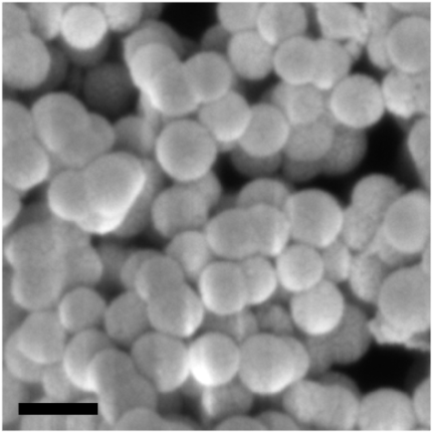


**A**

**B**

**Figure S2.** SEM images of HMSNs (A) and HMSNs-S-S-CPA-CytC-LA (B). Scale bar: 200 nm.


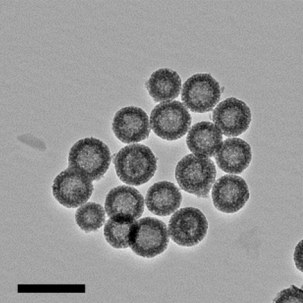

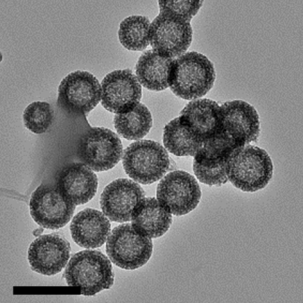

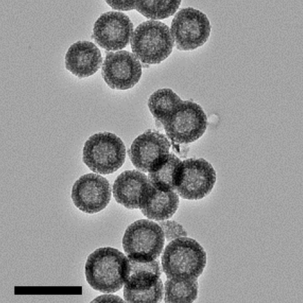

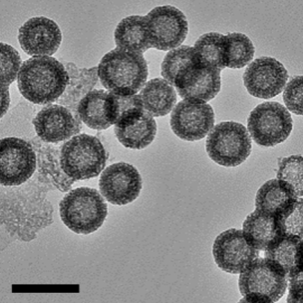

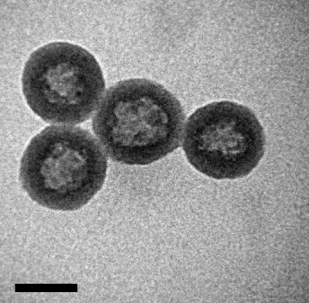

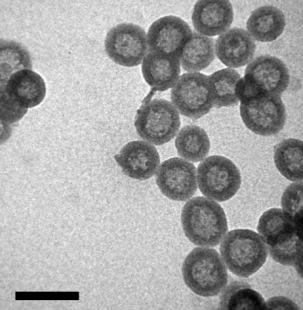

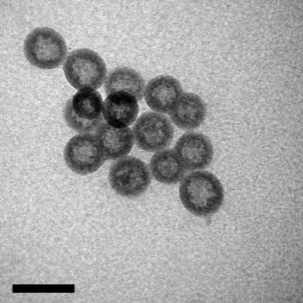

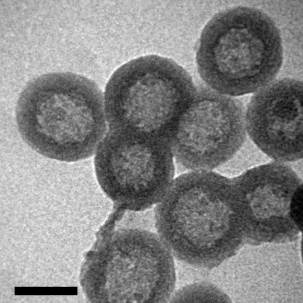


**A**

**B**

**C**

**D**

**E**

**F**

**G**

**H**

**Figure S3.** TEM images of HMSNs (A, B, C and D) and HMSNs-S-S-CPA-CytC-LA (E, F, G and H) nanoparticles. The scale bars were 200 nm for A, B, C, D, E and F, 100 nm for G and H.

**A B**







**Figure S4.** Particles size distributions of HMSNs (A) and HMSNs-S-S-CPA-CytC-LA (B) via dynamic light scattering (DLS) analysis.


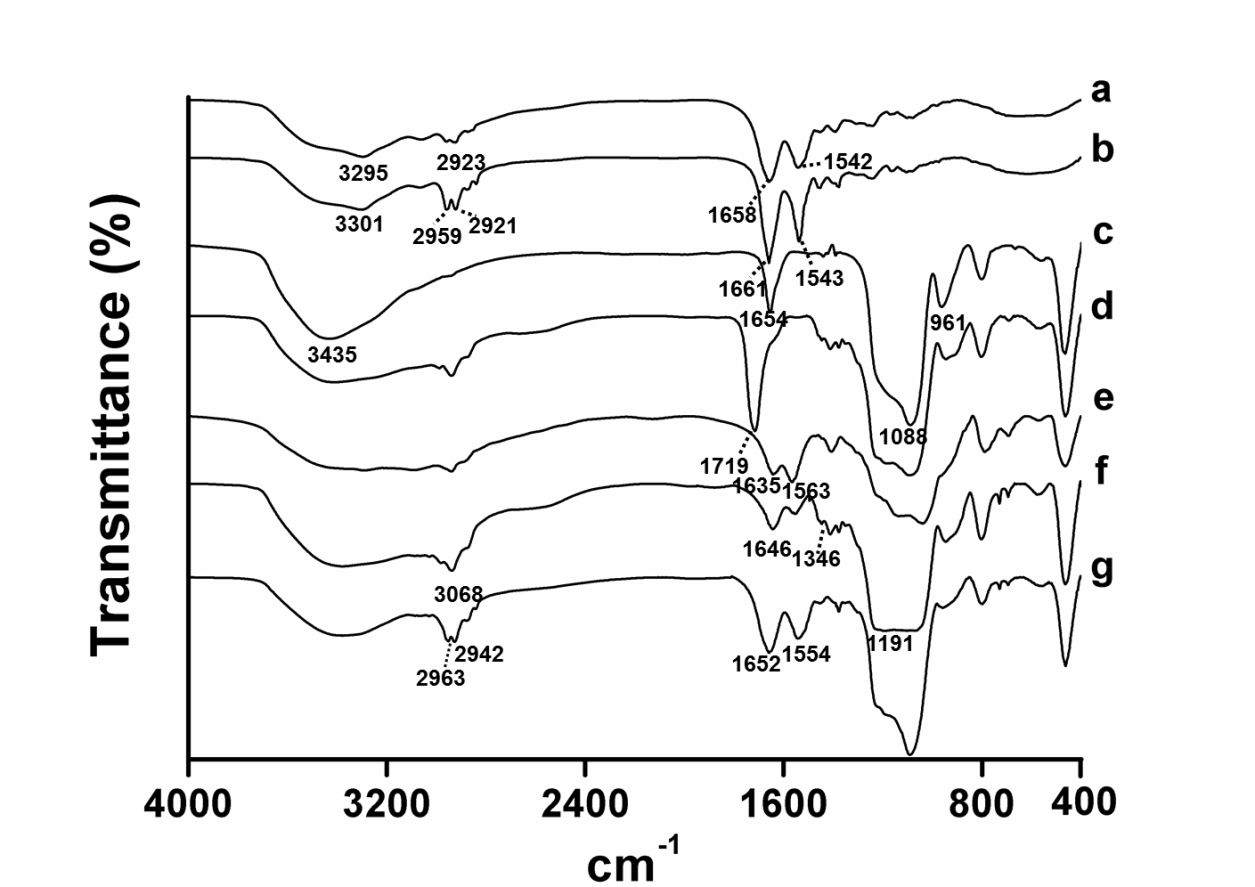


**Figure S5.** FTIR spectra of (a) CytC, (b) CytC-LA, (c) HMSNs, (d) HMSNs-COOH, (e) HMSNs-S-S-NH_2_, (f) HMSNs-S-S-CPA, and (g) HMSNs-S-S-CPA-CytC-LA.

**Figure S5** shows the FTIR spectra of the functionalization processes of HMSNs with various molecules. CytC displayed strong peaks at 1542 cm^-1^ and 1658 cm^-1^, which were attributed to amideⅠand amideⅡ groups in CytC molecules **(Figure S5, a)**. After conjugation with LA, no obvious differences in peaks between the spectra of CytC-LA and CytC were observed. However, the peak intensity of amide Ⅰ (1658 cm^-1^) and amide Ⅱ (1542 cm^-1^) groups slightly increased. Meanwhile, the peaks intensity at 2921 cm^-1^ and 2959 cm^-1^ obviously increased, which was attributed to the stretching of –CH– and –CH_2_– units in the conjugated LA molecules **(Figure S5, b)**. The results suggest that LA was successfully conjugated to CytC, which was consistent with a previous study [S1].

HMSNs displayed strong absorption signals at 961cm^-1^ and 1088 cm^-1^, which were attributed to the skeletal vibration of C-O bonds and the asymmetric stretching of Si-O-Si bridges. The weak peaks at 1654 cm^-1^ and 3435 cm^-1^ were assigned to physically adsorbed water molecules in HMSNs **(Figure S5, c)**. Compared to HMSNs, HMSNs-COOH displayed additional peaks at 1719 cm^-1^, which was assigned to the stretching vibration of C=O **(Figure S5, d)**. The results indicate the successful carboxylation of HMSNs.

After conjugation with cystamine dihydrochloride, the carboxyl group signal (around 1719 cm^-1^) disappeared in the spectrum of HMSNs-S-S-NH_2_ **(Figure S5, e)**. Moreover, additional peaks at 1635 cm^-1^ and 1563 cm^-1^ were observed, owing to the introduction of cystamine dihydrochloride molecules. The results demonstrate that disulfide bonds were covalently connected to HMSNs.

For HMSNs-S-S-CPA **(Figure S5, f)**, a new peak at 1346 cm^-1^ appeared, which was assigned to the B-O bond in phenylboronic acid molecules [S2]. Meanwhile, the peak at 1563 cm^-1^ (–NH_2_) significant decreased. It was related to the fact that the –COOH groups in 4-carboxyphenylboronic acid (CPA) molecules were reacted with the –NH_2_ groups on the surfaces of HMSNs. The intensity of peak at 1646 cm^-1^ increased due to the amide reaction. In addition, distinctive absorption peak at 3068 cm^-1^ was observed, which was attributed to the introduction of methyl groups deriving from 4-carboxyphenylboronic acid molecules. All above results provide direct evidence that CPA molecules were coupled to HMSNs-S-S-NH_2_. After further reaction with CytC-LA, new peaks at 2963 cm^-1^ and 2942 cm^-1^ were observed from the spectrum of HMSNs-S-S-CPA-CytC-LA (**Figure S5, g)**, which was assigned to the skeletal vibration of –CH– and –CH_2_– deriving from CytC-LA molecules. Besides, the intensity of peaks at 1652 cm^-1^ and 1554 cm^-1^ for amide Ⅰ and amide Ⅱ groups slightly increased. All results suggest that the HMSNs-S-S-CPA-CytC-LA was successfully synthesized.

**A B**




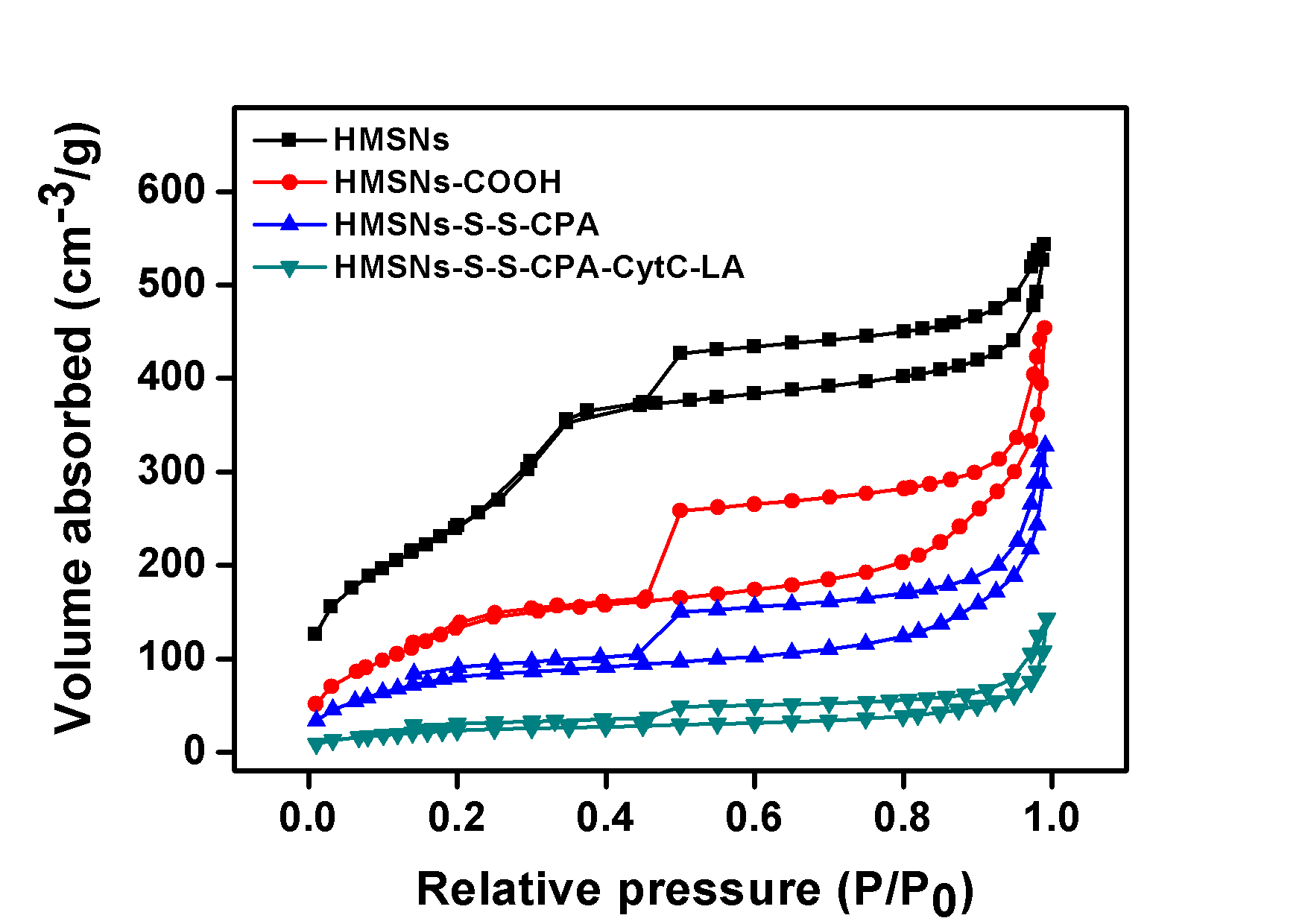


**Figure S6.** BET nitrogen adsorption/desorption isotherms (A) and BJH pore size distributions (B) of various HMSNs.


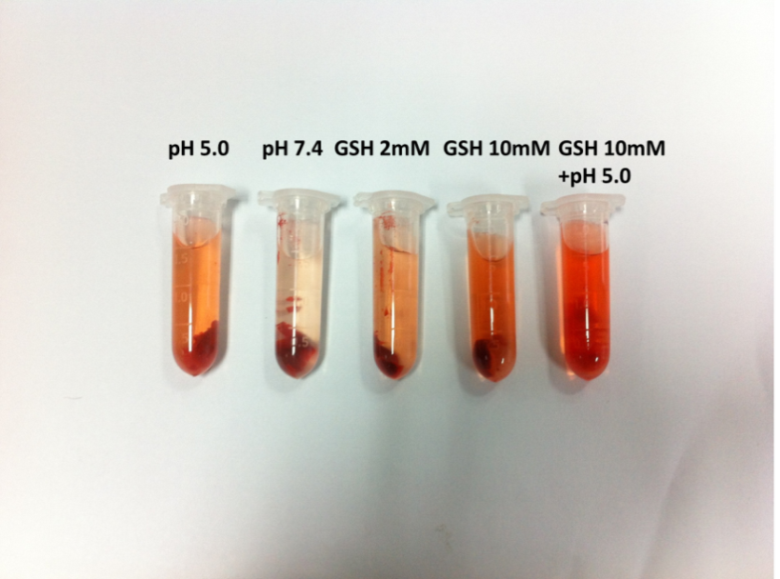


**Figure S7**. Optical images of DOX release from the HMSNs-S-S-CPA-CytC-LA@DOX system after incubation with various stimuli for 24 h.


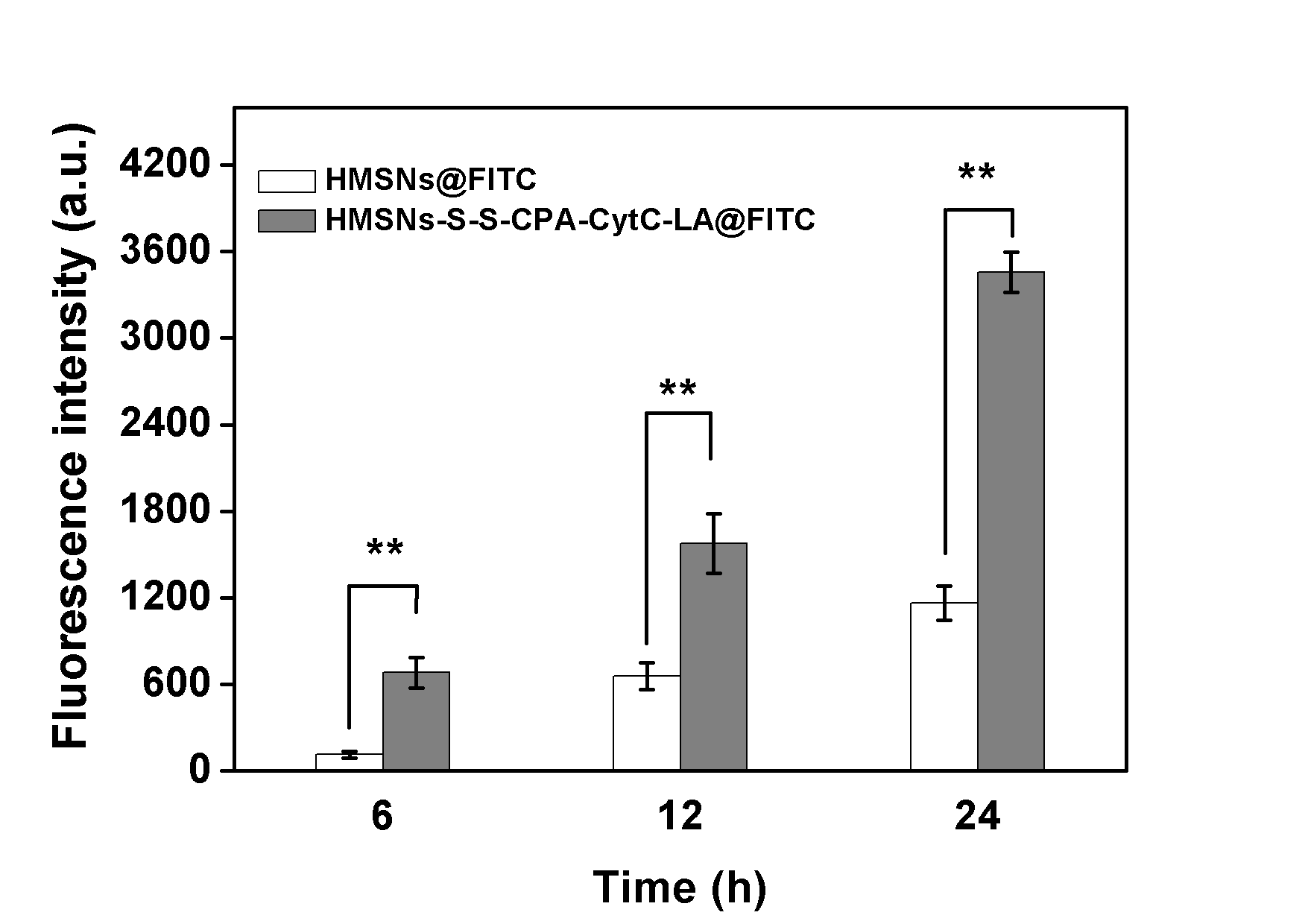


**Figure S8.** Quantitative fluorescence intensity analysis of FITC loaded nanoparticles within HepG2 cells after incubation with HMSNs@FITC and HMSNs-S-S-CPA-CytC-LA @FITC for 6, 12 and 24 h, respectively. The error bars represent mean ± SD (n = 3), **p < 0.01.


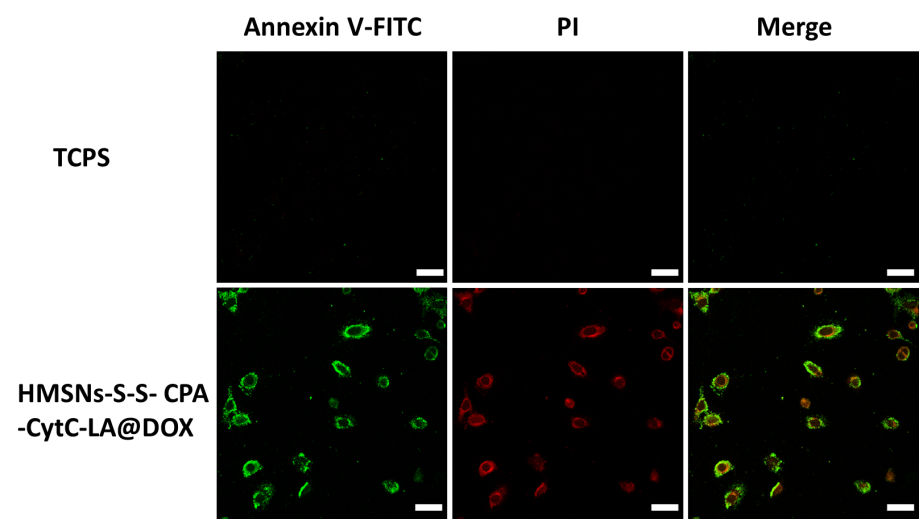


**Figure S9**. CLSM analysis the apoptosis of HepG2 cells after culture with HMSNs-S-S-CPA-CytC-LA@DOX for 24h with dual fluorescence staining of Annexin V-FITC/PI. Scale bars: 20 µm.


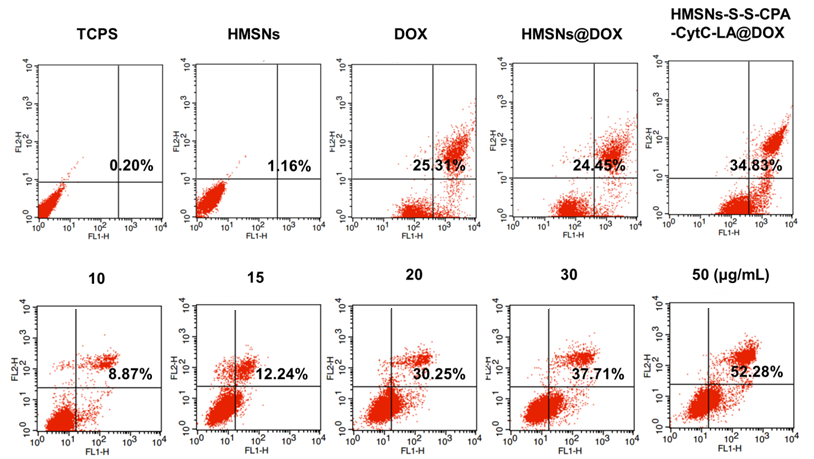


**Figure S10.** (A) Flow cytometry analysis the apoptosis of HepG2 cells after incubation with PBS (control), HMSNs (0.136 mg/mL), DOX (20 μg/mL), HMSNs@DOX (0.136 mg/mL), and HMSNs-S-S-CPA-CytC-LA@DOX (0.136 mg/mL) for 24 h; (B) Flow cytometry analysis the dose-dependent apoptosis of HepG2 cells after culture with HMSNs-S-S-CPA-CytC-LA@DOX for 24h.

**Table S1.** BET and BJH parameters of HMSNs before and after modified with different functional units.

| **Materials** | **BET**  **S_BET_(m^2^/g)** | **BET**  **V_P_(cm^3^/g)** | **BJH**  **W_BJH_(nm)** |
| --- | --- | --- | --- |
| HMSNs | 881.5411 | 0.763353 | 3.35553 |
| HMSNs-COOH | 652.7500 | 0.656735 | 3.18136 |
| HMSNs-S-S-CPA | 343.8025 | 0.405935 | 3.05325 |
| HMSNs-S-S-CPA-CytC-LA | 90.3512 | 0.176202 | / |

**Table S2.** Zeta-potential results of HMSNs before and after modifications with various functional units.

| **Materials** | **Zeta potential(mV)** |
| --- | --- |
| HMSNs | -23.6±4.21 |
| HMSNs-COOH | -30.9±7.34 |
| HMSNs-S-S-NH_2_ | 18.2±2.96 |
| HMSNs-S-S-CPA | 2.85±1.09 |
| HMSNs-S-S-CPA-CytC-LA | -24.3±4.61 |

**References**

[S1] Luo Z, Cai KY, Hu Y, Zhang BL, Xu DW. Cell-Specific Intracellular Anticancer Drug Delivery from Mesoporous Silica Nanoparticles with pH Sensitivity. Adv Healthc Mater. 2012;1:321-5.

[S2] Rodríguez-Cuamatzi P, Arillo-Flores O I, Bernal-Uruchurtu M I, et al. Theoretical and experimental evaluation of homo-and heterodimeric hydrogen-bonded motifs containing boronic acids, carboxylic acids, and carboxylate anions: Application for the generation of highly stable hydrogen-bonded supramolecular systems[J]. Crystal growth & design, 2005, 5(1): 167-175.
