# Supplementary material for: Phenylboronic acid-modified hollow silica nanoparticles for dual-responsive delivery of doxorubicin for targeted tumor therapy
Source: Regen Biomater. 2017 Jan 11;4(2):111–24. doi: 10.1093/rb/rbw045 (PMC6371689; doi:10.1093/rb/rbw045)
Supplement: Supplementary Data [file rbw045_Supp.zip › Responses to editor and reviewers.docx]

**Responses to the editor and reviewers**

*Reviewer#1:*

*C：This manuscript has been well-organized and written, in my opinion it can be accepted without change for publication.*

A: Thanks for your appreciation on our work.

*Reviewer#2:*

*C：In this manuscript, the authors reported a redox and pH dual-responsive drug delivery system based on hollow mesoporous silica nanoparticles (HMSNs) for targeting tumor therapy. The DOX loading HMSNs system could be specifically endocytosed by HepG2 cells and resulted in cell apoptosis and tumor growth inhibition with high efficiency. I would recommend this paper for publication in Regenerative Biomaterials, but some concerns are still needed to be addressed.*

A: Thanks for your interests in our work with instructive advices. Based on your suggestions, we revised the manuscript one by one as follows:

*Q1. The authors should provide the SEM images of HMSNs more clearly.*

A: Thanks for your kind suggestion. We provided SEM images of HMSNs with high resolution in the revised manuscript. Please see Figure 1 and Figure S2, as presented below.

**A**

**B**

**E**

**F**

**C**

**D**

**Figure 1.** Physical property characterizations: SEM and TEM images of HMSNs (A & B) and HMSNs-S-S-CPA-CytC-LA (D & E) nanoparticles with corresponding high resolution TEM images (C & F). The scale bars were 200 nm for A, B, D and E, 100 nm for C and F.

**A**

**B**

**Figure S2.** SEM images of HMSNs (A) and HMSNs-S-S-CPA-CytC-LA (B). Scale bar: 200 nm.

*Q2. For the cellular uptake, how about other tumor cells or normal cells? And the cell targeting property of HMSNs-S-S-CPA-CytC-LA should be further demonstrated by using different cells.*

A: Thank you for your kind suggestions. To investigate the cellular uptake and targeting property of *HMSNs-S-S-CPA-CytC-LA*, HepG2 (tumor cells) and HUVEC (normal cells) cells had been employed to co-culture with nanoparticles and evaluate its uptake efficiency, which detected by CLSM and FC, as shown in Figure 5. These results had suggested that HMSNs-S-S-CPA-CytC-LA@FITC was specifically and high efficiency endocytosed by tumor cells via a receptor-ligand recognition pathway, compared to normal cells. Please see page 21 lines 3-17, as marked with YELLOW backgrounds in the revised manuscript.

*Q3. Cell apoptosis analysis in vitro could be examined with the dual fluorescence of Annexin V-FITC/PI by CLSM.*

A: Thank you for your kind advice. We added the dual fluorescence of Annexin V-FITC/PI by CLSM in the revised supporting information as shown in Figure S9. Please see page 12 lines 2-3, page 23 lines 2-13, and Figure S9, as marked with YELLOW background in the revised manuscript.

**Figure S9**. CLSM analysis the apoptosis of HepG2 cells after culture with HMSNs-S-S-CPA-CytC-LA@DOX for 24 h with dual fluorescence staining of Annexin V-FITC/PI. Scale bars: 20 µm.
